# Risk prediction models for intracranial hemorrhage in acute ischemic stroke patients receiving intravenous alteplase treatment: a systematic review

**DOI:** 10.3389/fneur.2023.1224658

**Published:** 2024-01-05

**Authors:** Yaqi Hua, Chengkun Yan, Cheng Zhou, Qingyu Zheng, Dongying Li, Ping Tu

**Affiliations:** ^1^Department of Intensive Care Unit, The Second Affiliated Hospital of Nanchang University, Nanchang, China; ^2^School of Nursing, Nanchang University, Nanchang, China; ^3^Department of Post Anesthesia Care Unit, The Second Affiliated Hospital of Nanchang University, Nanchang, China

**Keywords:** ischemic stroke, tissue plasminogen activator, intracranial hemorrhage, models, systematic review

## Abstract

**Objectives:**

To identify and compare published models that use related factors to predict the risk of intracranial hemorrhage (ICH) in acute ischemic stroke patients receiving intravenous alteplase treatment.

**Methods:**

Risk prediction models for ICH in acute ischemic stroke patients receiving intravenous alteplase treatment were collected from PubMed, Embase, Web of Science, and the Cochrane Library up to April 7, 2023. A meta-analysis was performed using Stata 13.0, and the included models were evaluated using the Prediction Model Risk of Bias Assessment Tool (PROBAST).

**Results:**

A total of 656 references were screened, resulting in 13 studies being included. Among these, one was a prospective cohort study. Ten studies used internal validation; five studies used external validation, with two of them using both. The area under the receiver operating characteristic (ROC) curve for subjects reported in the models ranged from 0.68 to 0.985. Common predictors in the prediction models include National Institutes of Health Stroke Scale (NIHSS) (OR = 1.17, 95% CI 1.09–1.25, *p* < 0.0001), glucose (OR = 1.54, 95% CI 1.09–2.17, *p* < 0.05), and advanced age (OR = 1.50, 95% CI 1.15–1.94, *p* < 0.05), and the meta-analysis shows that these are independent risk factors. After PROBAST evaluation, all studies were assessed as having a high risk of bias but a low risk of applicability concerns.

**Conclusion:**

This study systematically reviews available evidence on risk prediction models for ICH in acute ischemic stroke patients receiving intravenous alteplase treatment. Few models have been externally validated, while the majority demonstrate significant discriminative power.

## Introduction

According to the latest global stroke data, approximately 12.2 million new stroke cases and 101 million existing stroke patients are reported worldwide each year, making it the second leading cause of death globally (1, 2). Ischemic strokes account for about 62.4–82.6% of these cases (1, 3). Revascularization methods for patients with acute ischemic stroke (AIS) include intravenous thrombolysis, intra-arterial thrombolysis, endovascular thrombectomy, and bridging thrombolytic therapy with endovascular thrombectomy (4, 5). The American Stroke Association (ASA) recommends intravenous thrombolysis with alteplase within 4.5 h of AIS onset as the first-line treatment (6). Alteplase is currently the only approved intravenous medication for AIS (7). However, its use can lead to complications such as hemorrhage, vascular edema, and seizures (8, 9). Intracranial hemorrhage (ICH) is the most severe complication, potentially resulting in prolonged hospital stays, increased medical costs, and a higher likelihood of disability and death (10). The one-year mortality rate associated of ICH patients is reported to be 52.2% (11). Therefore, the early recognition and management of ICH are crucial for AIS patients receiving intravenous alteplase. Numerous models for predicting ICH risk in AIS patients treated with intravenous alteplase have been developed worldwide. The purpose of this study is to thoroughly review and systematically summarize and compare these risk prediction models for ICH (including asymptomatic and symptomatic intracranial hemorrhage), aiming to enhance their construction and application in the management of AIS patients treated with intravenous alteplase.

## Methods and analysis

This research protocol is registered in the International Prospective Register of Systematic Reviews (PROSPERO) with the registration number CRD42023415649.

### Search strategy

A literature search was conducted in the following databases: PubMed, Embase, Web of Science, and the Cochrane Library. The search terms included “ischemic stroke,” “intracranial hemorrhage,” “alteplase,” and “factor.” Our complete search string can be found in Supplementary material S1. The search deadline was April 7, 2023.

### Eligibility criteria

The inclusion criteria for literature are: (1) type of research, either cohort study or case–control study; (2) participants, patients treated with alteplase after an acute ischemic stroke (AIS) who experienced intracranial hemorrhage, including asymptomatic and symptomatic intracranial hemorrhage; (3) studies providing relevant content on model construction and validation. The exclusion criteria are: (1) lack of practicality of predictive factors in clinical practice; (2) inability to obtain the full text; (3) duplicate publications.

### Literature screening and data extraction

Two researchers independently screened the literature and conducted cross-checks. Disagreements were resolved by consulting a third researcher. Information from the included literature was extracted regarding basic information, model performance, model composition, etc., as shown in Tables 1, 2.

**Table 1:** Basic characteristics and effectiveness evaluation of the included models.

Study	Country	Research type	Modeling sample size		Modeling method	Verification model method	Criteria for ICH	Model performance	
			Total	Outcome events				AUC (Modeling/Verification)	Calibration test method
Cucchiara et al., 2008 (12)	Multiple countries	Retrospective cohort study	1,205	72	Logistic regression	-	CT	0.69/−	-
Mazya et al., 2012 (13)	Multiple countries	Retrospective cohort study	13,908	-	Logistic regression	Internal	CT or MRI	0.71/0.69	H-L test
Menon et al., 2012 (14)	America	Retrospective cohort study	10,242	496	Logistic regression	Internal + External	-	0.71/0.68	H-L test
Strbian et al., 2012 (15)	Multiple countries	Retrospective cohort study	974	68	Logistic regression	External	CT	0.77/−	H-L test
Lokeskrawee et al., 2017 (16)	Thailand	Retrospective cohort study	1,172	249	Logistic regression	Internal	CT	0.75/0.76	H-L test
Cappellari et al., 2018 (17)	Italy	Retrospective cohort study	12,030	647	Logistic regression	Internal	-	0.699/0.739	H-L test
Erdur et al., 2018 (18)	Italy	Retrospective cohort study	1,336	53	Logistic regression	External	ECASS-III	0.72/0.69	-
Wu et al., 2020 (19)	China	Retrospective cohort study	131	16	Logistic regression	Internal	NCCT	0.956/0.985	Calibration plots
Zhou et al., 2020 (20)	China	Retrospective cohort study	233	33	Logistic regression	Internal	CT	0.828/0.801	H-L test and Calibration plots
Xie et al., 2021 (21)	China	Retrospective cohort study	462	20	Logistic regression	Internal	CT	0.878/0.877	-
Weng et al., 2022 (22)	China	Retrospective cohort study	387	31	Logistic regression	Internal + External	CT	0.887/0.776	Calibration plots
Xu et al., 2022 (23)	China	Retrospective cohort study	345	45	Machine learning	Internal	CT or MRI	0.795/0.703	BHFDR
Yang et al., 2022 (10)	China	Prospective cohort study	257	45	Logistic regression	External	NCCT or MRI	0.859/0.839	H-L test

“-” means not stated in the paper. ICH, Intracranial Hemorrhage; AUC, Area Under Curve; CT, Computed Tomography; MRI, Magnetic Resonance Imaging; H-L test, Hosmer-Lemeshow test; NCCT, Non-Contrast Computed Tomography; ECASS-III, European-Australasian Acute Stroke Study; BHFDR, Benjamini Hochberg false discovery rate.

**Table 2:** Predictors and stratification methods included in the study.

Study	Number of factors	Predictors	Risk factor assignment/Risk stratification method
Cucchiara et al., 2008 (12)	4	Age > 60,NIHSS > 10 points, GLU > 150 mg/dL, PLT < 150,000/mm^3^	All four factors are 1 point. The incidence of ICH increases with the increase of scores: 0 points, 2.6%; 1 point, 9.7%; 2 points, 15.1%; ≥ 3 points, 37.9%.
Mazya et al., 2012 (13)	9	NIHSS, GLU, SBP, Age, Weight, OTT, Aspirin or aspirin plus clopidogrel, Hypertension	The odds ratio (OR) in the logistic regression model was used to assign values to each factor. The total score ranges from 0–12, 0–2 low risk, 3–5 average risk, 6–8 medium risk, ≥9 high risk.
Menon et al., 2012 (14)	6	Age, NIHSS, SBP, GLU, Asian race, Male sex	Based on the β coefficient in the logistic regression model Weighting each predictor. The total score ranges from 45–101.
Strbian et al., 2012 (15)	5	GLU at admission, Early infarct signs, (hyper)Dense cerebral artery sign, Age > 75,NIHSS	Based on the β coefficient in the logistic regression model Weighting each predictor. 1 point: GLU at admission 8.1 ~ 12.0 mmol/L, Early infarct signs, (hyper)Dense cerebral artery sign, age > 75, NIHSS≥10 points;2 points: GLU at admission12.0 mmol/L. The total score ranges from 0–6.
Lokeskrawee et al., 2017 (16)	6	Valvular heart diseases, Aspirin, SBP before thrombolysis≥140 mmHg, NIHSS, PLT < 250,000/mm^3^,Use of intravenous antihypertensive drugs during thrombolysis	The odds ratio (OR) in the logistic regression model was used to assign values to each factor. 1 point: SBP before thrombolysis≥140 mmHg,PLT < 250,000/mm^3^,use of intravenous antihypertensive drugs during thrombolysis; 1.5 points: Aspirin; 2 points: valvular heart diseases, NIHSS 10-19points;3 points: NIHSS > 20 points.
Cappellari et al., 2018 (17)	10	SBP, Age, OTT, NIHSS, GLU, Aspirin, aspirin plus clopidogrel, Anticoagulant with INR ≤ 1.7, Infarct signs, (hyper)Dense cerebral artery sign	Based on logistic regression analysis, select the predictive factors and form a nomogram through weighted scores
Erdur et al., 2018 (18)	5	Age, NIHSS, GLU at admission, and treatment with medium or high-dose statins	The risk factors are scored according to the β coefficients in the logical regression analysis, and developing a risk calculator.
Wu et al., 2020 (19)	4	CDS,CSVD,NIHSS≥13 points, OTT ≥ 180 min	The nomogram was created by assigning a graphic preliminary score to each of the predictors with a point ranging from 0 to 100, which was then summed to generate a total score, finally converted to an individual probability (from 0 to 100%) of ICH.
Zhou et al., 2020 (20)	3	Atrial fibrillation, NIHSS, GLU at admission	The nomogram was created by assigning a graphic preliminary score to each of the predictors with a point ranging from 0 to 100, which was then summed to generate a total score, finally converted to an individual probability (from 0 to 100%) of ICH.
Xie et al., 2021 (21)	4	NIHSS, OTT, NLR, Cardioembolism	Based on logistic regression analysis, select the predictive factors and form a nomogram through weighted scores
Weng et al., 2022 (22)	4	Smoke, NIHSS, BUN/Cr, NLR	The risk factors are scored according to the β coefficients in the logical regression analysis, and visualized using nomograms.
Xu et al., 2022 (23)	4	Triglyceride, Lpa, NIHSS, hemoglobin	Algorithm assigns values to four prediction factors to form a prediction model
Yang et al., 2022 (10)	4	Early infarct signs, NIHSS, Uric acid, AGR	Based on logistic regression analysis, select the predictive factors and form a nomogram through weighted scores. The score range of the nomograms is 0–11 points

NIHSS, National Institutes of Health Stroke Scale; GLU, glucose; PLT, platelet count; ICH, Intracranial Hemorrhage; SBP, Systolic Blood Pressure; OTT, Onset to treatment time; INR, International normalized ratio; CDS, Chronic disease scale; CSVD, Cerebral small vascular disease; NLR, Neutrophil-to-lymphocyte ratio; Lpa, Lipoprotein(a); AGR, Albumin-to-globulin ratio.

### Statistical analysis

A meta-analysis was performed using Stata (version 13.0). The relationship between risk factors and ICH in patients with AIS receiving intravenous alteplase treatment was explored through the odds ratio (OR) and corresponding 95% confidence interval (CI). Heterogeneity between studies was detected using the Q-test, and appropriate effect models were selected. A sensitivity analysis was conducted by sequentially excluding studies. If Begg’s test and/or Egger’s test (*p* < 0.05) indicated publication bias, the trim-and-fill method was employed for reassessment.

### Bias risk assessment

Two researchers used the Prediction Model Risk of Bias Assessment Tool (PROBAST) (24) to evaluate the included study’s bias risk and consulted a third researcher in cases of disagreement. PROBAST evaluates the risk of bias and the applicability of the model across four domains: participants, predictors, outcomes, and analysis.

### Predictive performance

The predictive performance of the model was evaluated through discrimination and calibration. The discrimination index is the Area Under the Curve (AUC), with an AUC ≥ 0.7 indicating good discrimination of the model, and externally tested AUC being more convincing. Calibration indicators include the Hosmer-Lemeshow test and calibration plots. A model is considered well-fitted if the Hosmer-Lemeshow test yields a *p*-value >0.05, or if the calibration plot’s slope is close to 1.

## Results

### Screening process and results

**Figure 1:**
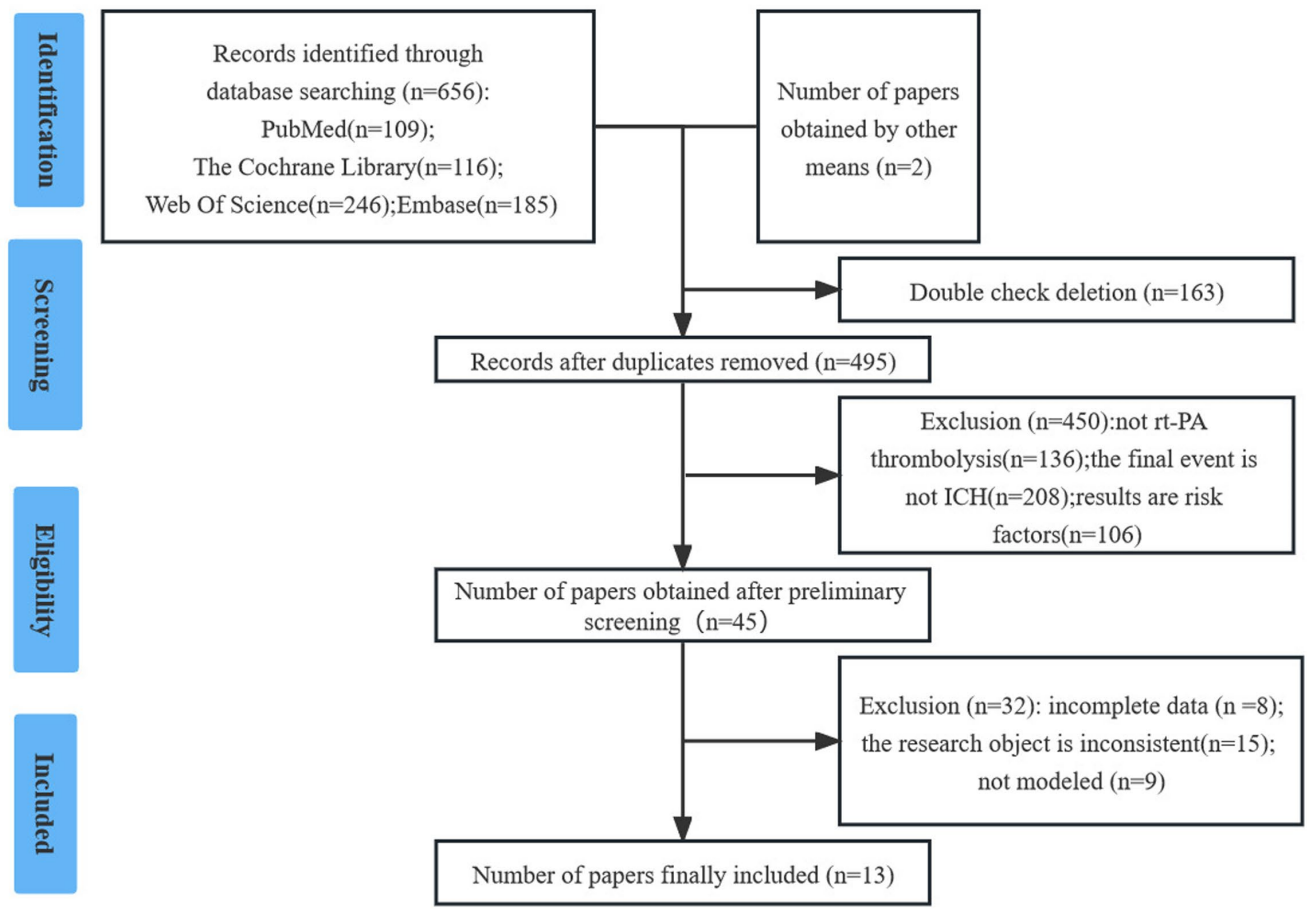
PRISMA flow diagram of study selection process.

The researchers initially identified 657 studies. After screening, 13 studies (10, 12–23) were included in the final analysis. Details are provided in Figure 1.

### General information on included studies

A total of thirteen risk prediction models for intracranial hemorrhage (ICH) in patients with acute ischemic stroke (AIS) receiving intravenous alteplase treatment were included. Of these, three were conducted in multiple countries (12, 13, 15), six in China (10, 19–23), two in Italy (17, 18), one in America (14), and one in Thailand (16). Regarding the type of research, one was a prospective cohort study (10), and the others were retrospective. The earliest included publication year was 2008 (12), with four articles published in the past three years (10, 21–23). All studies defined participants as patients treated with alteplase for AIS; six specified participants as 18 years or older (10, 17, 20–23), demonstrating high homogeneity in the studies (Table 1).

### Modeling and verification

In these studies, the modeling sample size ranged from 131 to 13,908, with the incidence of ICH varying from 3.97 to 21.25%. For modeling methods, one study (23) employed machine learning (ML), while the remaining 12 studies (10, 12–22) used single-factor analysis combined with logistic regression. Regarding model validation, two studies (14, 22) used both internal and external validation, seven studies (13, 16, 17, 19–21, 23) used internal validation, and three studies (10, 15, 18) used only external validation (Table 1).

### Predictors and assignment

The number of predictive factors in the models varied, with the maximum being 10 (17) and the minimum three (25). Common predictors of ICH in AIS patients treated with intravenous alteplase were NIHSS (*n* = 13), glucose (*n* = 7), and advanced age (*n* = 6). For risk factor assignment, 12 studies (10, 12–22) utilized OR values or logistic regression β coefficients to assign weights to predictors. One study (23) used machine learning for weight allocation, as detailed in Table 2.

### Meta-analysis for risk factors

A meta-analysis was conducted for NIHSS, glucose, and advanced age. Some studies did not provide effective OR values and 95% CIs, hence they were excluded from the meta-analysis. The results indicated that NIHSS, glucose, and advanced age are independent risk factors for ICH in AIS patients treated with intravenous alteplase (Table 3).

**Table 3:** The meta-analysis for risk factors.

Factors	No. of studies	Effects model	OR (95%CI)	*P*	Heterogeneity	
					*I*^2^ (%)	*P*_Q_
NIHSS	9 (10, 13, 14, 16, 18–22)	REM	1.17 (1.09–1.25)	< 0.0001	84.2	0.001
Glucose	4 (13, 14, 18, 20)	REM	1.54 (1.09–2.17)	0.015	93.9	0.001
Advanced age	3 (13, 14, 18)	REM	1.50 (1.15–1.94)	0.003	80.9	0.005

REM, random-effects model.

**Figure 2:**
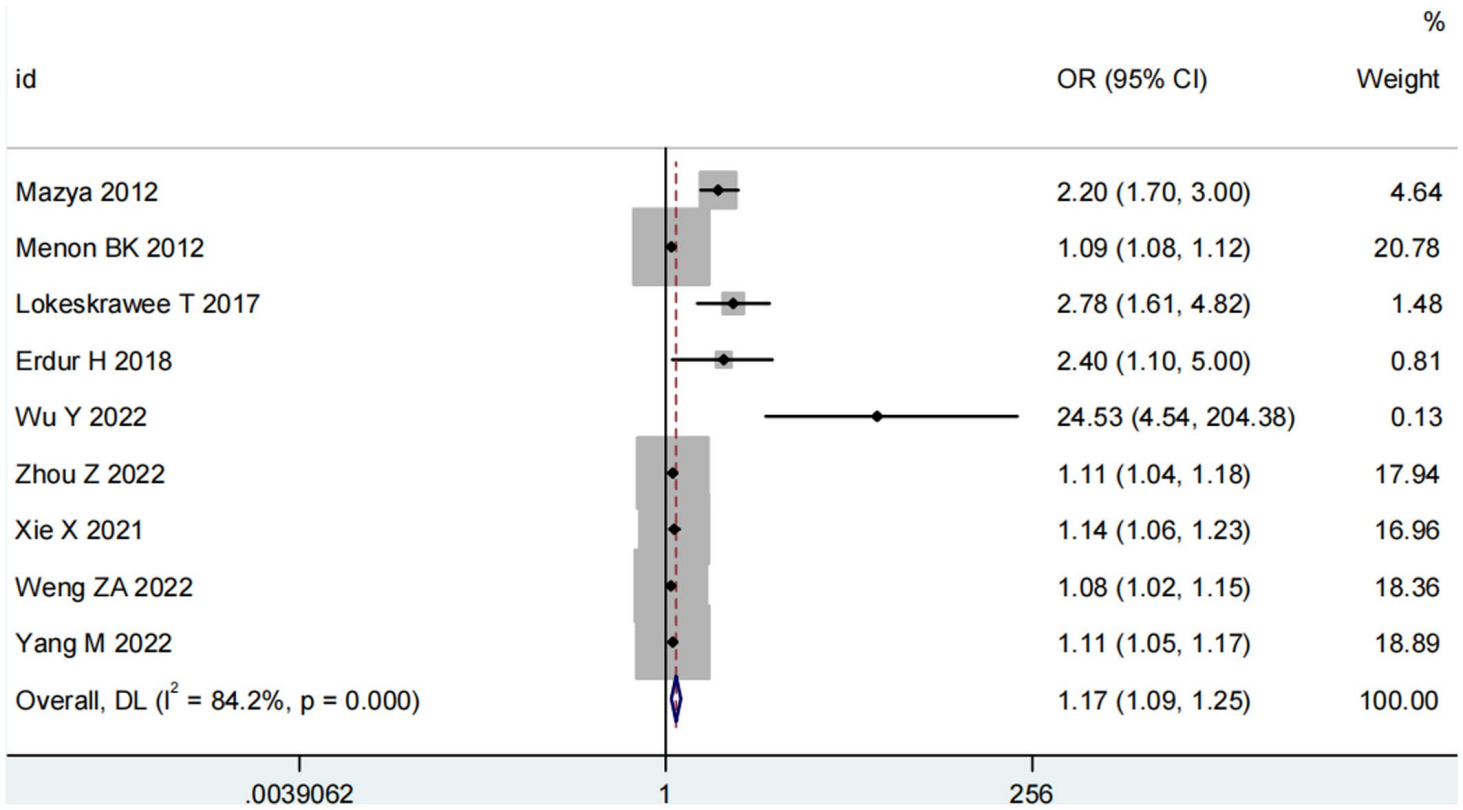
Meta-analysis of NIHSS on ICH in patients receiving intravenous alteplase therapy after AIS.

**Figure 3:**
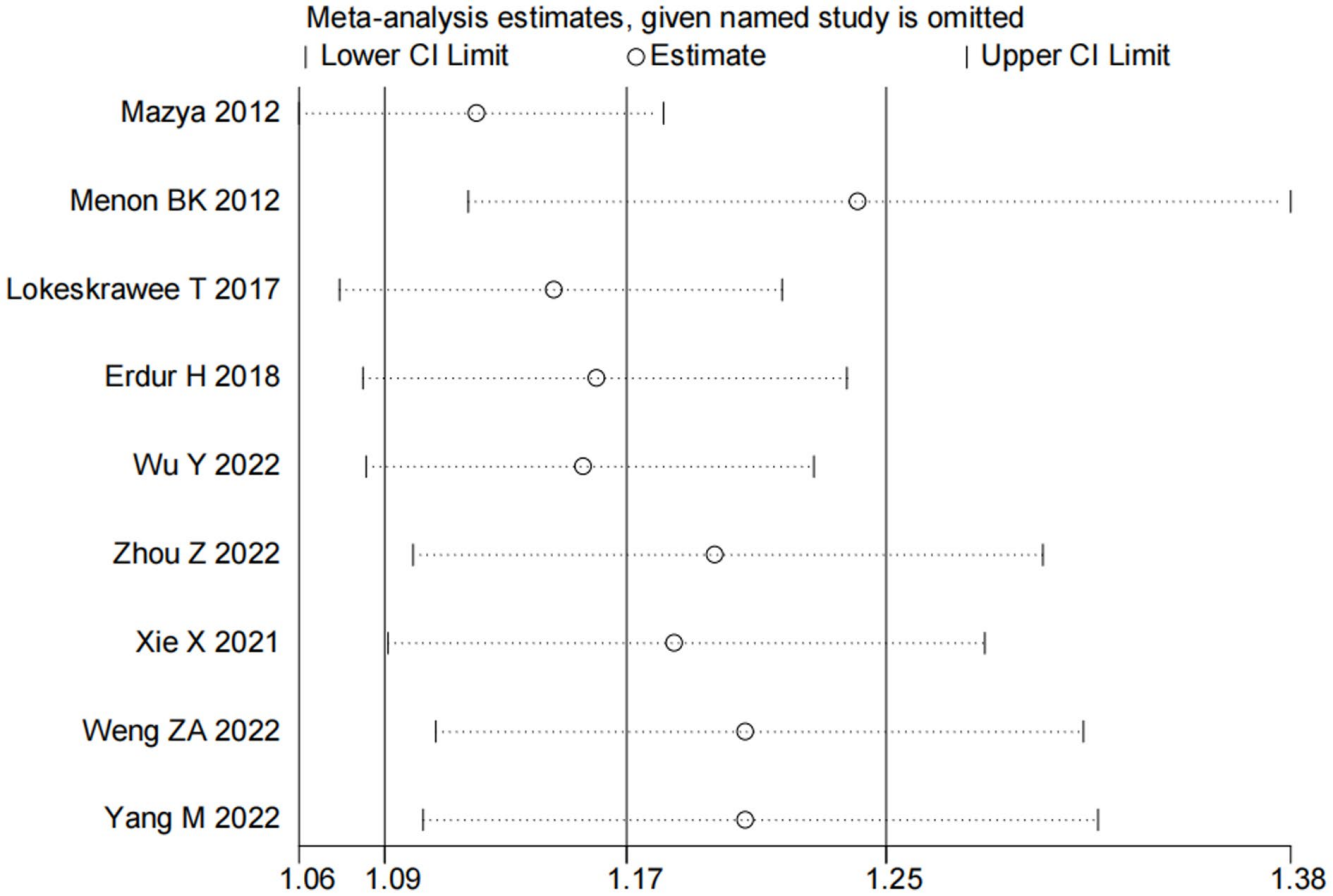
Sensitivity analysis for the association between NIHSS and ICH.

**Figure 4:**
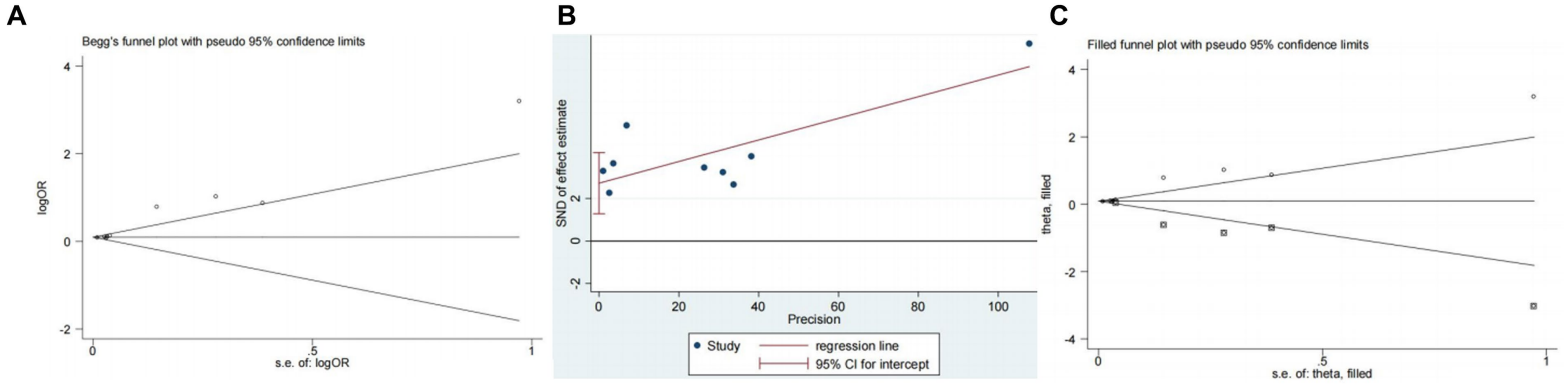
Plots for publication bias test in meta-analysis for the association between NIHSS and ICH. **(A)** Begg’s funnel plot; **(B)** Egger’s publication bias plot; **(C)** Filled funnel plot.

Nine studies (10, 13, 14, 16, 18–22) examined the impact of NIHSS on ICH in patients treated with intravenous alteplase post-AIS (heterogeneity: *p* = 0.001, I^2^ = 84.2%). There was a significant difference between the groups (95% CI: 1.09–1.25, *p* < 0.0001; Figure 2). A subgroup meta-analysis based on sample size was conducted due to significant differences in sample sizes included. The meta-analysis revealed NIHSS as an independent risk factor for ICH in all patient subgroups (Table 4). Sensitivity analysis indicated that excluding any study did not significantly alter the meta-analysis results (Figure 3). Begg’s test (*p* < 0.05) and Egger’s test (*p* < 0.05) suggested publication bias (Figures 4A,B). The trim-and-fill method was used to correct for publication bias. After three iterations using the linear method, the adjusted OR remained significant (OR = 1.096, 95% CI 1.013–1.185, *p* < 0.05), indicating the reliability of the results (Table 4).

**Table 4:** Subgroup meta-analysis of NIHSS based on sample size.

Cut-off value	No. of studies	No. of patients	Effects model	HR (95%CI)	*P*	Heterogeneity	
						*I*^2^ (%)	*P*_Q_
≥10,000	2 (13, 14)	24,150	REM	1.53 (0.77–3.03)	< 0.001	95.7	0.000
≥1,000	2 (16, 18)	2,508	FEM	2.64 (1.70–4.12)	< 0.001	0	0.758
≥100	5 (10, 19–22)	1,470	REM	1.11 (1.06–1.18)	< 0.001	64.9	0.023

REM, random-effects model; FEM, fixed-effects model.

**Figure 5:**
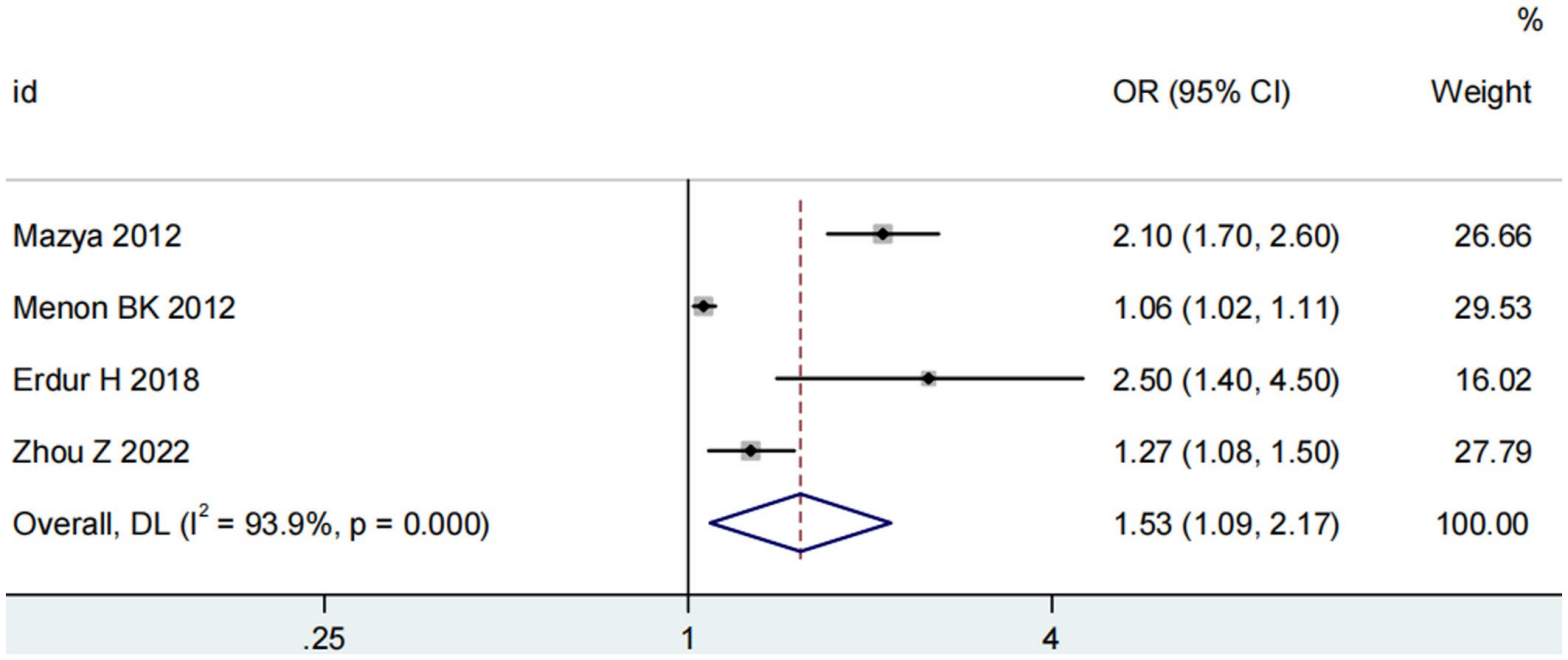
Meta-analysis of glucose on ICH in patients receiving intravenous alteplase therapy after AIS.

**Figure 6:**
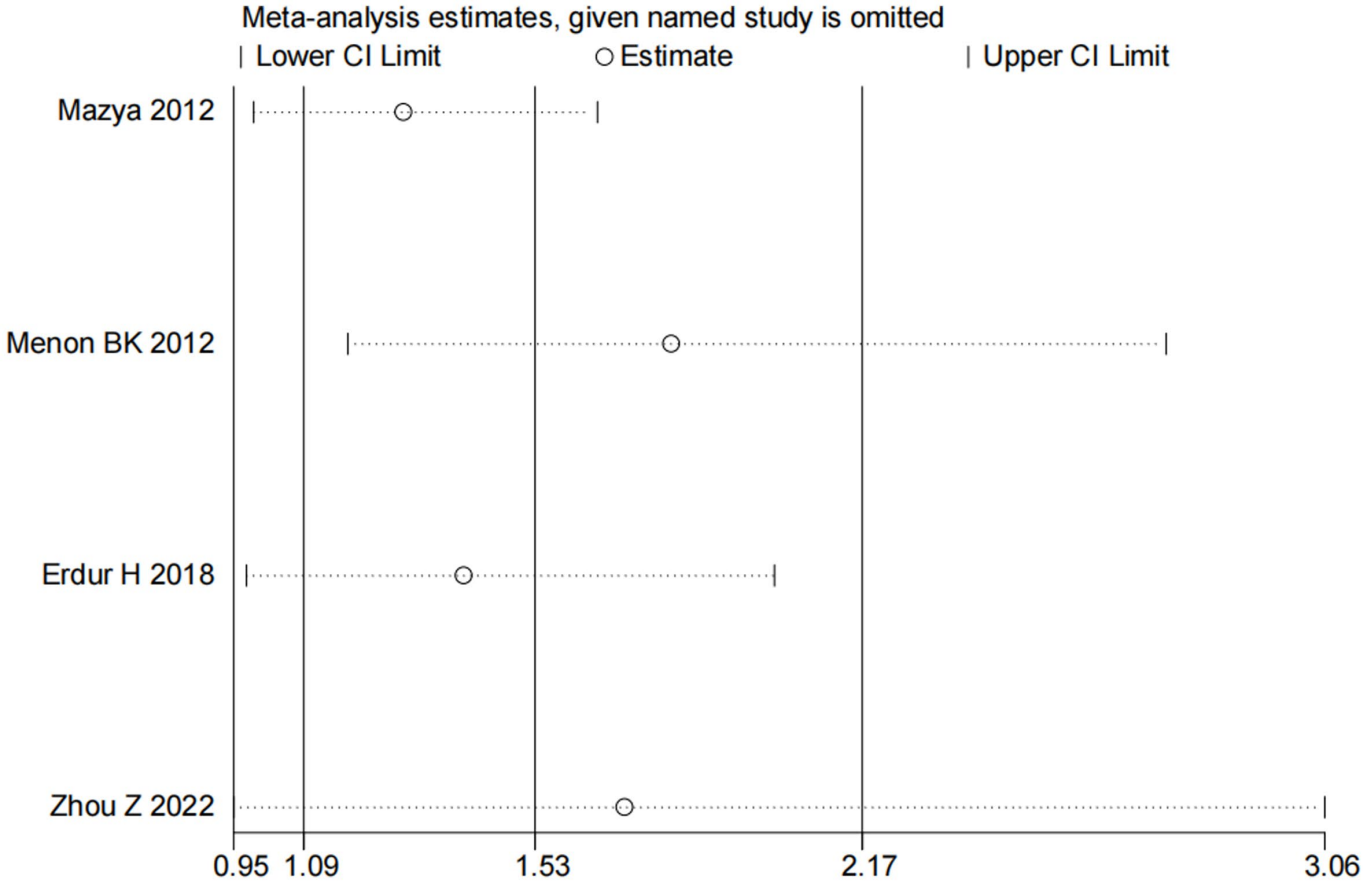
Sensitivity analysis for the association between glucose and ICH.

**Figure 7:**
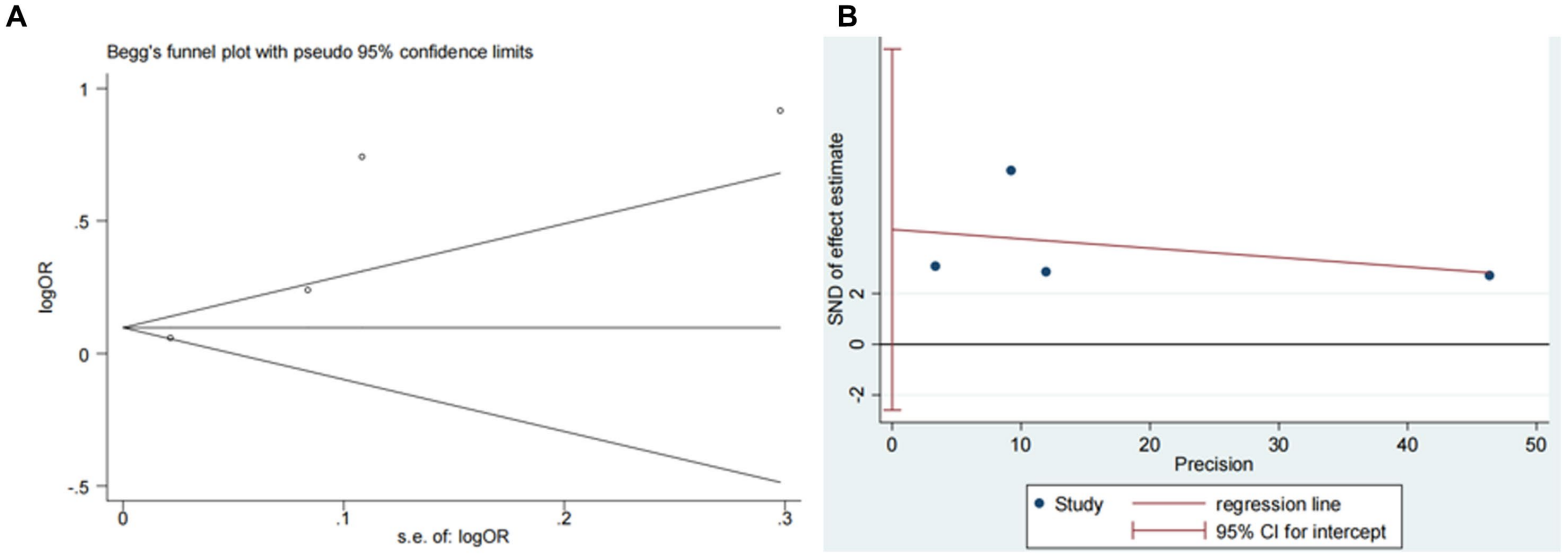
Plots for publication bias test in meta-analysis for the association between glucose and ICH. **(A)** Begg’s funnel plot; **(B)** Egger’s publication bias plot.

Four studies (13, 14, 18, 20) explored the impact of glucose on ICH in patients receiving intravenous alteplase post-AIS (heterogeneity: *p* = 0.001, I^2^ = 93.9%). A significant difference was observed between groups (95% CI: 1.09–2.17, *p* < 0.05; Figure 5). Sensitivity analysis confirmed the robustness of the result (Figure 6). Begg’s test (*p* > 0.05) and Egger’s test (*p* > 0.05) showed no publication bias (Figures 7A,B).

**Figure 8:**
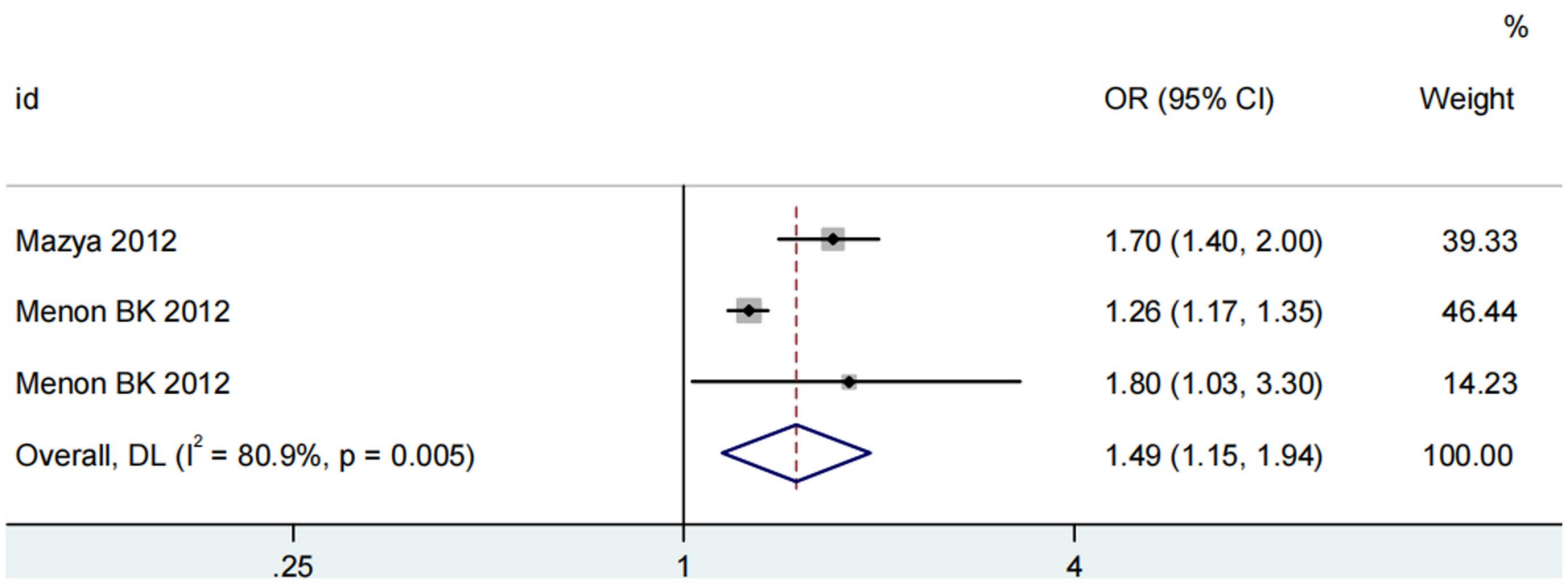
Meta-analysis of advanced age on ICH in patients receiving intravenous alteplase therapy after AIS.

**Figure 9:**
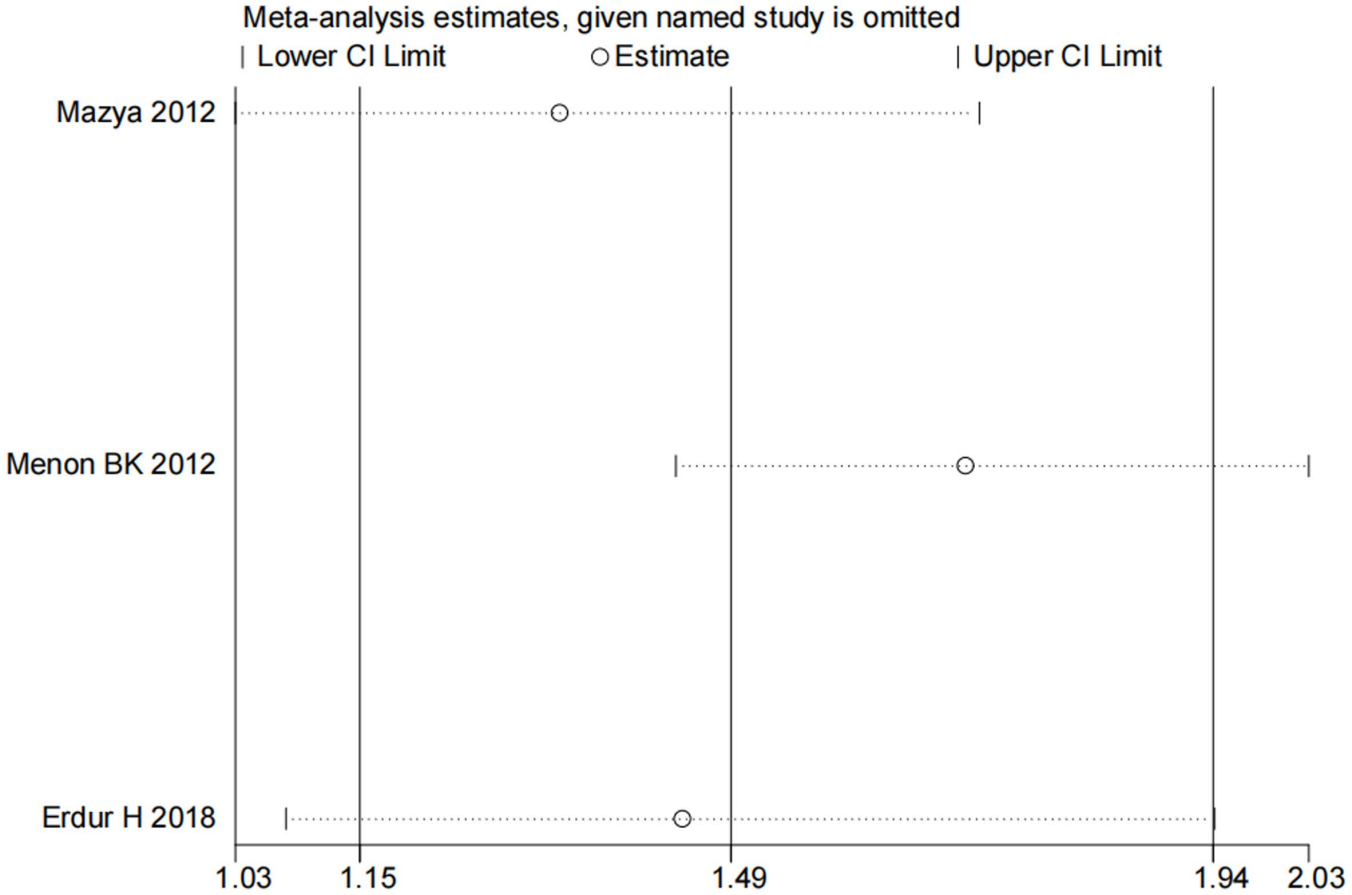
Sensitivity analysis for the association between advanced age and ICH.

**Figure 10:**
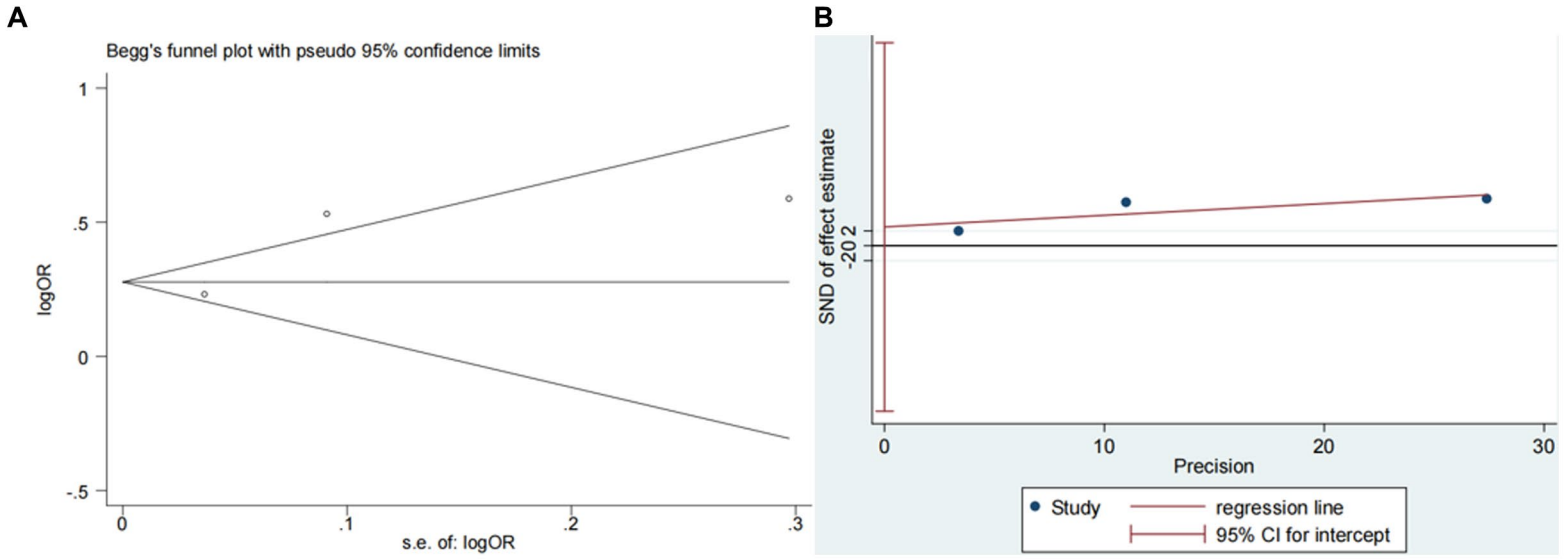
Plots for publication bias test in meta-analysis for the association between advanced age and ICH. **(A)** Begg’s funnel plot; **(B)** Egger’s publication bias plot.

Three studies (13, 14, 18) assessed the impact of advanced age on ICH in patients treated with intravenous alteplase post-AIS (heterogeneity: *p* = 0.005, I^2^ = 80.9%). The difference was statistically significant (95% CI: 1.15–1.94, *p* < 0.05; Figure 8). Sensitivity analysis showed that excluding any study did not change the results (Figure 9). Begg’s test (*p* > 0.05) and Egger’s test (*p* > 0.05) indicated no significant publication bias (Figures 10A,B).

### Methodological quality evaluation

Among the thirteen pieces of literature included, the bias risk assessment results indicated a high risk for all, primarily due to participant selection and statistical analysis aspects. However, the applicability evaluation results for all the studies were considered low-risk (Table 5).

**Table 5:** Risk of bias assessment results included in the model (PROBAST).

Study	Risk of Bias Assessment					Applicability evaluation			
	Participants	Predictors	Outcome	Analysis	Total	Participants	Predictors	Outcome	Total
Cucchiara et al., 2008 (12)	3	1	1	3	3	1	1	1	1
Mazya et al., 2012 (13)	3	1	1	3	3	1	1	1	1
Menon et al., 2012 (14)	3	1	2	3	3	1	1	1	1
Strbian et al., 2012 (15)	3	1	1	3	3	1	1	1	1
Lokeskrawee et al., 2017 (16)	3	1	1	3	3	1	1	1	1
Cappellari et al., 2018 (17)	3	1	2	3	3	1	1	1	1
Erdur et al., 2018 (18)	3	1	1	3	3	1	1	1	1
Wu et al., 2020 (19)	3	1	1	3	3	1	1	1	1
Zhou et al., 2020 (20)	3	1	1	3	3	1	1	1	1
Xie et al., 2021 (21)	3	1	1	3	3	1	1	1	1
Weng et al., 2022 (22)	3	1	1	3	3	1	1	1	1
Xu et al., 2022 (23)	3	1	1	3	3	1	1	1	1
Yang et al., 2022 (10)	1	1	1	3	3	1	1	1	1

1 = low risk; 2 = unclear risk; 3 = high risk.

### Predictive performance evaluation

Regarding discrimination, four studies reported AUCs less than 0.7 during model establishment or validation, suggesting suboptimal model performance (12–14, 17). Strbian et al. (15, 18) reported a modeling AUC of 0.77. Five studies (16, 19–21, 23) achieved AUCs greater than 0.7 in both modeling and internal validation. Wu et al. constructed models with AUCs exceeding 0.95 (19), and two studies (10, 22) reported AUCs greater than 0.7 in both modeling and external validation. For calibration, six studies (10, 13–17) used the Hosmer-Lemeshow test with all results indicating well-fitting models (*p* > 0.05). The calibration slopes in two studies (19, 22) were close to 1, and the model by Zhou et al. (20) showed good performance by both criteria. Based on these two indicators, the model by Weng ZA et al. (22) is considered to have exceptional performance.

## Discussion

Overall, the development of risk prediction models for intracranial hemorrhage (ICH) in patients with acute ischemic stroke (AIS) receiving intravenous alteplase treatment is still in its early stages. The research spans a considerable time frame, with a primary focus on America, Thailand, Italy, and China. Most of the models lack external validation and have not yet been implemented clinically.

### Discussion on overall bias risk

The risk of bias in prediction models is closely associated with participants, predictors, outcomes, and analysis. All 13 articles included in our study exhibited a high risk of bias (10, 12–23). According to PROBAST, data from prospective cohort studies are considered more reliable than those from retrospective cohort studies (24). However, our study included only one prospective cohort study (10). PROBAST also stipulates that to avoid overfitting, the modeling sample size should include more than 20 events per variable (EPV), and the validation sample size should comprise at least 100 subjects (26). Seven studies did not meet this standard, increasing the risk of model bias (10, 15, 19–23). Regarding the treatment of independent variables, several studies (12–14, 16–20) simplified continuous variables into categorical ones, reducing information efficiency and potentially lowering model performance. As for missing data, three studies (12, 13, 18) failed to address this issue, while the others (10, 14–17, 19–23) excluded samples with missing data. Most studies (10, 12–22) selected factors through uni-variate analysis, and five (13, 15, 17, 18, 21) modified the significance level in this analysis. However, uni-variate analysis can overlook col-linearity between independent variables, leading to the selection of inappropriate factors. Therefore, the Transparent Reporting of a Multivariate Prediction Model for Individual Prognosis or Diagnosis (TRIPOD) recommends adjusting the significance level of uni-variate analysis or employing stepwise regression (27). In terms of model performance, ten studies (10, 13–17, 19, 20, 22, 23) reported both discrimination and calibration. Discrimination is measured by the Area Under Curve (AUC). Calibration is typically assessed using Hosmer-Lemeshow tests and calibration plots, though six studies only used Hosmer-Lemeshow tests (10, 13–17). The *p*-value in Hosmer-Lemeshow tests does not fully represent model calibration (27); calibration plots are recommended. Two studies (19, 22) utilized calibration plots, and one study (20) used both methods. Model performance may display optimistic bias due to over-fitting, underscoring the need for effective validation methods. Seven studies (13, 16, 17, 19–21, 23) only conducted internal validation, with four (13, 17, 20, 23) using the less efficient split-sample method. It is advised to use bootstrap sampling or cross-validation for internal validation; two studies (16, 21) used bootstrap sampling, and one (19) combined randomized splitting with bootstrap sampling. PROBAST (24) indicates that the absence of external validation in predictive model development leads to a high overall bias risk. Only five studies (10, 14, 15, 18, 22) employed external validation, but one (15) did not report the AUC for external validation. Regarding model applicability: All studies defined participants as patients treated with alteplase for AIS, aligning with our study design. Additionally, the definition, assessment, and timing of evaluating predictive factors in all models are consistent with our study. Lastly, the determination of ICH in all included studies matches our study, confirming the excellent overall applicability of the studies included (10, 12–23).

### Prediction factor analysis

The models included in this study encompass a variety of predictive factors, including general data, disease-related information, biochemical indicators, and imaging results. While each model comprises different predictive factors, common elements are present. Notably, NIHSS, glucose, and advanced age were found to be strongly associated with intracranial hemorrhage (ICH) in patients with acute ischemic stroke (AIS) receiving intravenous alteplase treatment. Meta-analysis confirms that these factors are independent risk factors, aligning with the findings of numerous related studies (28–30). The National Institutes of Health Stroke Scale (NIHSS) is widely used for assessing stroke severity and is endorsed by the American Stroke Association (ASA) guidelines as an effective tool for emergency departments to evaluate stroke severity (31, 32). All models included in our study featured NIHSS, and meta-analysis validated that a higher NIHSS score is an independent risk factor for ICH. Whiteley WN et al. conducted a meta-analysis of 55 articles on ICH risk factors in AIS patients treated with intravenous alteplase, concluding that a higher NIHSS score is a risk factor for ICH (33). Teekaput et al.’s retrospective study on 725 AIS patients who received alteplase treatment demonstrated that the incidence of ICH in patients with a higher baseline NIHSS was 1.9 times that of the control group (11). Patients with AIS and a high baseline NIHSS typically have larger infarct areas and more extensive vascular damage, increasing their susceptibility to ICH following alteplase treatment (32, 34). Therefore, careful consideration of treatment methods, enhanced monitoring, and proactive intervention are crucial for patients with high baseline NIHSS scores to effectively prevent ICH. Glucose is another independent predictor of ICH, consistent with previous research findings. Hyperglycemia is common in the acute phase of AIS, often resulting from stress-induced increases in cortisol and catecholamine levels following ischemic injury. Elevated glucose levels are associated with stroke severity and adverse outcomes (35, 36). Advanced age is also a significant predictor. Dong S. et al.’s meta-analysis of 25 cohort studies established that advanced age is an independent predictor of ICH in AIS patients (37). This is particularly relevant for patients over 80 years old receiving alteplase treatment (38). As patients age, their overall physical condition deteriorates, the prevalence of cardiovascular diseases rises, blood vessel elasticity decreases, and brain parenchyma undergoes degenerative changes, all of which contribute to a higher risk of ICH after thrombolysis (38, 39).

### Advantages and limitations

#### Advantages

(1) This study systematically integrates recent risk prediction models for intracranial hemorrhage (ICH) in patients with acute ischemic stroke (AIS) receiving intravenous alteplase treatment, emphasizing the modeling method, model performance, predictive factors, and factor assignment. (2) It employs PROBAST to evaluate the quality of these models, analyze risk sources, and provide a reference for future research. (3) The study enhances the reliability of its conclusions by supplementing quantitative analysis through meta-analysis.

#### Limitations

(1) The study is limited to literature published in English. (2) There are variations in the study populations and the standards for defining ICH among the included studies. (3) Some models lack validation, necessitating further research to verify their applicability.

## Conclusion

In summary, there has been a steady increase in the number of risk prediction models for ICH in AIS patients treated with intravenous alteplase. However, the performance of these models varies. The 13 models included in this study present a high risk of bias due to statistical analysis issues, but they generally exhibit a low risk regarding overall applicability, which is beneficial for the early screening of high-risk patients. Due to the high overall bias risk in the included studies, it is not advisable to directly apply these predictive models in clinical practice. Medical professionals should aim to facilitate the application and generalization of existing models through external validation involving multiple centers and large samples or conduct large-sample prospective research to develop new predictive models in accordance with TRIPOD and PROBAST guidelines.

## Data availability statement

The original contributions presented in the study are included in the article/Supplementary material, further inquiries can be directed to the corresponding author.

## Author contributions

YH and PT conceived the study idea, designed the study, conducted database searches, and wrote the manuscript. CY and CZ interpreted the data and provided additional relevant information. QZ and YH analyzed the quality of each included study and confirmed the analysis results. DL and PT oversaw the methodology, and reviewed and revised the manuscript. All authors have read and approved the final manuscript.

## Supplementary material

Supplementary Material 1 Complete search strategy.

Supplementary Material 2 Subgroup meta-analysis of NIHSS on ICH in patients receiving intravenous alteplase therapy after AIS.
