# Supplementary material for: Risk prediction models for intracranial hemorrhage in acute ischemic stroke patients receiving intravenous alteplase treatment: a systematic review
Source: Front Neurol. 2024 Jan 5;14:1224658. doi: 10.3389/fneur.2023.1224658 (PMC10799340; doi:10.3389/fneur.2023.1224658)
Supplement: Supplementary Material 1 — Complete search strategy. [file Data_Sheet_1.pdf]

Search strategy

(Ischemic Stroke OR cerebral infarct OR cerebral embolism OR cerebral ischemia OR Poststroke OR cerebral infarction OR Bain Ischemia OR Cerebral Infarction OR Lacunar Stroke OR Lacunar Infarct OR Brain Thrombosis OR Cerebral Thrombus OR Brain Infarctions OR Infarction, Brain OR Brain Infarct) AND (Cerebrum Hemorrhage OR Intracerebral Hemorrhage OR Encephalorrhagia OR intracranial hemorrhage OR Hemorrhage transformation OR Hemorrhage OR bleeding OR hemorrhagic transformation) AND (thrombolysis OR thrombolytic OR alteplase OR tissue plasminogen activator OR rt-PA OR rtPA) AND (Factor OR model OR Nomogram)
