# Supplementary figures and images for: Risk prediction models for intracranial hemorrhage in acute ischemic stroke patients receiving intravenous alteplase treatment: a systematic review
Source: Front Neurol. 2024 Jan 5;14:1224658. doi: 10.3389/fneur.2023.1224658 (PMC10799340; doi:10.3389/fneur.2023.1224658)

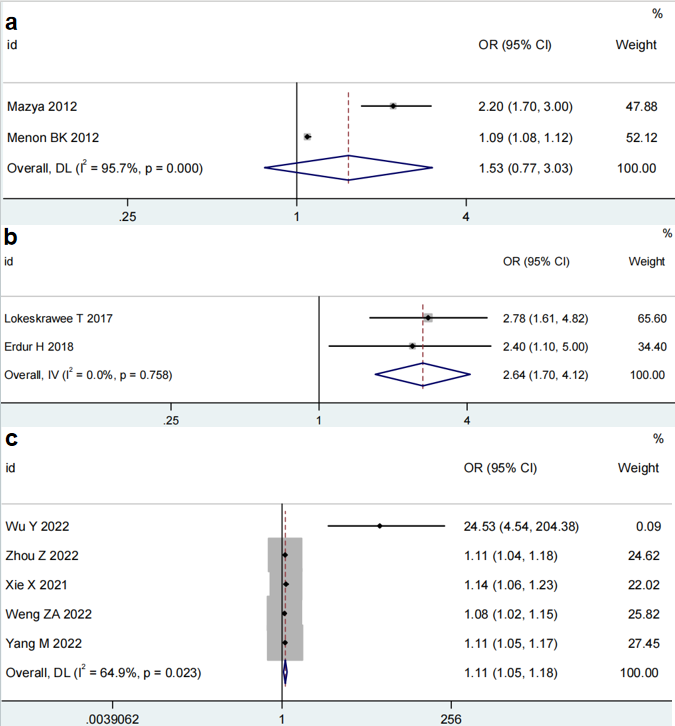

Supplement: Supplementary Material 2 — Subgroup meta-analysis of NIHSS on ICH in patients receiving intravenous alteplase therapy after AIS. [file Image_1.TIF]
